# Corpus Callosum Microstructural Tract Integrity Relates to Longer Emotion Recognition Reaction Time in People with Schizophrenia

**DOI:** 10.3390/brainsci12091208

**Published:** 2022-09-08

**Authors:** Tom Burke, Christina Gleeson, Laurena Holleran, David Mothersill, Jessica Holland, Laura Costello, Ruán Kane, Declan P. McKernan, Derek W. Morris, John P. Kelly, Aiden P. Corvin, Brian P. Hallahan, Colm McDonald, Gary Donohoe

**Affiliations:** 1 School of Psychology, National University of Ireland Galway, H91 TK33 Galway, Ireland; 2 Neuroimaging, Cognition, and Genomics Centre, National University of Ireland Galway, H91 TK33 Galway, Ireland; 3 Pharmacology & Therapeutics and Galway Neuroscience Centre, National University of Ireland Galway, H91 W5P7 Galway, Ireland; 4 Neuropsychiatric Genetics Research Group, Department of Psychiatry, Institute of Molecular Medicine, Trinity College Dublin, D02 R590 Dublin 2, Ireland; 5 Department of Psychiatry, Clinical Science Institute, National University of Ireland Galway, H91 TK33 Galway, Ireland

**Keywords:** social cognition, schizophrenia, white matter tract integrity, corpus callosum, emotion recognition, reaction time, symptom severity, diffusion-tract imaging, fractional anisotropy, interhemispheric processing

## Abstract

**Objective**: Schizophrenia is a complex functionally debilitating neurodevelopmental disorder, with associated social cognitive impairment. Corpus Callosum (CC) white matter tracts deficits are reported for people with schizophrenia; however, few studies focus on interhemispheric processing relative to social cognition tasks. This study aimed to determine if a relationship between the CC and social cognition exists. **Method:** In this cross-section study, a sample of n = 178 typical controls and n = 58 people with schizophrenia completed measures of mentalising (Reading the Mind in the Eyes), emotion recognition outcome and reaction time (Emotion Recognition Test), and clinical symptoms (Positive and Negative Symptom Scale), alongside diffusion-based tract imaging. The CC and its subregions, i.e., the genu, body, and splenium were the regions of interest (ROI). **Results:** Reduced white matter tract integrity was observed in the CC for patients when compared to controls. Patients performed slower, and less accurately on emotion recognition tasks, which significantly and negatively correlated to the structural integrity of the CC genu. Tract integrity further significantly and negatively related to clinical symptomatology. **Conclusions:** People with schizophrenia have altered white matter integrity in the genu of the CC, compared to controls, which relates to cognitive deficits associated with recognising emotional stimuli accurately and quickly, and severity of clinical symptoms.

## 1. Introduction

Schizophrenia is a neurodevelopmental disorder affecting approximately 1 in 300 people worldwide according to the World Health Organisation in 2022. Individuals diagnosed with schizophrenia have an increased risk of early mortality, broad cognitive impairment, and altered psychosocial functioning, compared to the general population [1]. According to the National Institute of Mental Health (NIMH) in 2021, the international prevalence rates are reportedly between 0.33–0.75%. There are various contributing factors to the development of schizophrenia, including genetic predisposition and polygenic risk [2], neuroinflammation [3], decreased synaptic spine density [4], cannabis use [5], psychosocial influences and reduced stress resilience [6], adverse childhood experiences and trauma [7], diminished anti-inflammatory response alongside reduced lipid metabolism [8], mitochondrial stress resilience [9], and gene–environment interaction effects [10]. In line with this, the diathesis–stress model may be a potential hypothesis accounting for the overall development of the disorder [11].

Schizophrenia is characterised by clinical symptoms, which significantly impact on everyday functioning [12]. Symptoms vary from person to person, though people with schizophrenia tend to experience a mixture of positive symptoms (i.e., delusions and hallucinations), negative symptoms (i.e., disorganized thought and behaviour, lack of motivation, restricted emotional awareness and apathy, social withdrawal, depression, and agitation), and cognitive impairment [13]. Cognitive deficits have been observed across multiple domains, including executive function, memory, attention, and social cognition [14], which correlate with functional neuroimaging studies [7,15,16,17].

Social cognition is a cognitive domain, which relates to several processes. These include individual and contextual perception and interpretation of behaviour [18], face, expression, and emotion recognition [19], as well as inferring the mental states of others [20]. These fundamental processes allow individuals to interpret, understand, and adapt to social situations on a behavioural level through shared attention and fundamental processes, such as gaze-orientation [21], and then respond appropriately to social cues and interactions in their environment [18]. Although aspects of social cognition are strongly linked to specified brain regions, such as the orbitofrontal cortex, anterior cingulate cortex, temporoparietal junction, and medial prefrontal cortex, no singular anatomical region is responsible for all aspects of social cognitive functioning, in line with a network-based understanding of this cognitive domain [22,23,24,25,26]. It has been well established that people with schizophrenia have impaired social cognition [27], though how that relates to their clinical syndrome, white matter integrity, and speed of processing emotionally salient stimuli is less well known. Social cognitive functioning is an important domain for consideration for people with schizophrenia, as it is a reliable predictor of occupational functioning and independent living capacity [28], even in early psychosis [29], and to consider its relationship with white matter tracts not only informs us about neuroanatomical differences in schizophrenia but also the impact that has on clinical profiles and social cognitive outcomes. Functionally, reduced social cognitive ability directly relates to reduced social and occupational functioning [29] and reduced community-based integration [30] and leads to malfunctioning social exchanges [31].

There is substantial evidence of structural brain changes in schizophrenia. Altered white matter microstructure is commonly measured in terms of fractional anisotropy (FA), which reflects diffusion in tissue, with lower FA often taken to indicate reduced anatomical integrity [32]. Reduced FA has been associated with lower cognitive performance both in healthy individuals and patients diagnosed with schizophrenia [33,34]. In schizophrenia, reduced FA is correlated with more global cognitive functions, such as poorer processing speed, working memory, and executive functioning [35,36]. Clinical symptoms of schizophrenia have also been shown to be negatively associated with WM tracts frontotemporal regions [37,38], as well as cerebellar regions [39]. However, the association between lower FA and cognitive deficits does not appear to be accounted for by variation in clinical symptom severity [34,39].

Brain regions involved in aspects of social cognition, such as emotion perception and recognition, relate to both hemispheres and include the fusiform face area, which is a specialised brain region responsible for the recognition of faces [25], located in the inferior temporal lobe. Face perception is a more right hemisphere dominant process [40,41], with volumetric decrease in the right fusiform gyrus reported for people with schizophrenia [42] and reduced event related potentials when perceiving faces [43]. Notwithstanding these right-hemisphere specific findings, Nazeri and colleagues [44] report more left-lateralized decreased FA in a study of white matter integrity of forty-four participants with schizophrenia, compared with typical controls, with additional studies highlighting lower WM integrity being associated with lower non-social cognitive domains, such as executive function [35], working memory [45], verbal memory [37], verbal and visual learning [39,46], and motor functioning [47]. Although there is growing evidence showing the relationship between white matter integrity and cognitive function, there is a paucity of research investigating the relationship to social cognitive outcomes. More specifically, while many of the aforementioned studies look at hemisphere-specific tracts, little to no studies have investigated the relationship between social cognitive outcomes and the corpus callosum (CC), which connects both hemispheres, and individually it has been identified as being impaired in people with schizophrenia [48]. Typically, the left hemisphere is more associated with language function [49], while the right hemisphere shows more evidence as dominant for emotion processing [50], yet tasks of social cognition typically require high functional ability in both these domains. Following surgical resection of the CC through corpus callosotomy typically for individuals with intractable epilepsy, for example, individuals are reported to have reduced capacity to interpret and identify emotion [51,52]. As such, interhemispheric tract integrity should be an area investigated specifically in relation to interhemispheric cognitive domains, such as social cognition.

The CC itself can be further segmented into the genu, body, and splenium (anterior to posterior, respectively). In particular, Subramaniam and colleagues [53] identified specific localised regions of impairment in the CC, which included the genu and splenium. Further findings from DTI studies have shown reduced WM integrity of the CC and its relationship with reduced cognitive function [54]. Astroglia density has been shown to be abnormal in the anterior region of the CC for people with schizophrenia, which may relate to specific social cognitive deficits [55], with some studies showing pharmacological intervention improving in WM integrity within the genu of the CC [56]. Studies have also reported associations between emotion recognition [54], clinical symptoms [48], and perspective taking [12], particularly within the genu, splenium, and body of the CC. Findings in the literature have been moderately inconsistent, however, and most have been more focused on broader cognition domains, unlike this study, which focuses on individual facets of emotion recognition and reaction time, the relationship with structural integrity of the CC and its subregions, and clinical symptomology. Consequently, this study hypothesises that individuals with schizophrenia will have reduced FA within the CC and its anatomical subregions compared with typical controls. We further hypothesise based on the literature that reduced FA in the CC will relate to higher clinical symptoms as a proxy for syndrome burden and severity and lower social cognitive outcomes [12,48,54,57].

## 2. Materials and Methods

### 2.1. Participant Information

Following ethical approval, a total of n = 236 participants were recruited to take part in this study. Of those recruited, there were n = 178 typical control participants, or ‘healthy controls’, and a total of 58 people with a diagnosis of schizophrenia (see Table 1 for patient and typical control demographics). Patients were recruited from outpatient departments and mental health facilities, such as day centres and day hospitals based in Ireland. Before the commencement of the study, participants were presented with an information sheet and were required to provide written consent.

### 2.2. Inclusion and Exclusion Criteria

All participants were required to be aged between 18–65 years. Inclusion criteria for typical controls included having no prior mental health condition/other general medical condition, no prior antipsychotic medication use, and no 1st degree relative diagnosed with schizophrenia or schizoaffective disorder. Patient inclusion criteria included having a previously established clinical diagnosis of schizophrenia or schizoaffective disorder, as outlined by the *Diagnostic and Statistical Manual of Mental Disorders* (DSM-IV [58]), and confirmed by the *Structured Clinical Interview for DSM-IV* (SCID [59]). Further inclusion criteria for patients included being clinically stable regarding medication and outpatient status when the study took place. Patients also were omitted if they had a comorbid psychiatric disorder. Broader exclusion criteria for both groups included having a history of an acquired brain injury, including loss of consciousness, intellectual disability (as measured by an obtained IQ score of under 70), or a history of substance abuse six months prior to study commencement. Patients and controls were omitted if they had contraindications for undergoing MRI scanning, such as the presence of a metal implant device or individuals susceptible to claustrophobia. Furthermore, individuals were not included in both cohorts if they had a history of a neurological disorder, such as epilepsy.

### 2.3. Materials and Procedure

#### MRI Procedure

Diffusion magnetic resonance imaging (MRI) was gathered for this project. An initial MRI scan was completed on all participants before disseminating diffusion data. The standard structural MRI scan was completed using a 3T Philips Achieva MR scanner. The scanner is located in the Centre for Advanced Medical Imaging at St. James’ Hospital in Dublin 8, Ireland. Each participant underwent a structural whole-brain MRI scan, which followed a pre-determined acquisition sequence, including three-dimensional T1-weighted images using a ‘Fast Field Echo’ pulse sequence with a spatial resolution of 1 mm^3^. The scan ‘Repetition Time’ (TR) was 8.5 milliseconds (ms), and ‘Echo Time’ (TE) was 3.9 milliseconds (ms). The ‘Inversion Time’ from the time elapsed between pulses was 1060 milliseconds (ms) with a ‘Flip Angle’ of 8°. The acquisition sequence was obtained in millimetres over a distance (field of view) of 256 × 256 × 160 mm^3^, and the acquisition time was 7 min and 30 s total. Foam padding was used to preserve a secured head position for the duration of the MRI scan, and participants were supplied with headphones to dampen noise interference.

### 2.4. ENIGMA DTI Protocol

To extract all of the variables using diffusion techniques, this study employed the utilization of the ENIGMA DTI protocol (https://enigma.ini.usc.edu/protocols/dti-protocols/) (last accessed 1 September 2022). The ENIGMA protocol was generated through an international consortium involving worldwide researchers in the field of genetics, neurology, and psychiatry. The working group involved in the ENIGMA cohort for schizophrenia contains 29 groups/sites from 14 countries and hosts 4322 participants in total, with a breakdown of 2359 healthy participants and 1963 people who have a schizophrenia diagnosis. Both groups in the sample were well matched on age. Healthy participants, defined as ‘typical controls’, had an average age of 36.14 (M = 36.14) within an age range of 18 years to 86 years. Of the participants with a schizophrenia diagnosis, the average age was 36.22 (M = 36.22), with an age range of 18 to 77 years. Of the participants with a schizophrenia diagnosis, the average age of onset recorded was 23 years, while the average age of duration of the disorder was 14 years.

The ENIGMA DTI pre-processing of diffusion images involved corrections and quality control (QC) procedures. This study fulfilled the ENIGMA procedures by the inclusion of pre-processing and QC steps involving eddy current echo-planar imaging corrections, which can both result in image distortion during diffusion imaging [60]. Tensor fitting was also included as a QC step. All DTI pre-processing and QC recommendations can be found at https://enigma.ini.usc.edu/protocols/dti-protocols/ (last accessed 1 September 2022). Using the DTI protocol outlined, the primary analysis of FA was established, and the following regions were extracted as specific regions of interest: the CC, the genu of the CC; the body of the CC; the splenium of the CC, with ‘Average FA across tracts’ extracted as a standardised variable [34].

### 2.5. Cognitive and Clinical Assessments

#### Cambridge Neuropsychological Test Automated Battery (CANTAB^TM^) Emotion Recognition Task

The CANTAB emotion recognition task (ERT [61]) is a widely used measure of emotion recognition in people with disorders, such as schizophrenia, autism, and depressive disorders. The ERT takes approximately 6–10 min to complete. A fixation cross appears on a screen, followed by an image appearing for a time of 200 ms. The task involves participants viewing a modified computer-based image of a person’s face, which displays a specific emotion and ranges in intensity between 1 and 15, in which 15 represents strongest intensity of a particular emotion. Following the image appearing on the screen, participants are presented with six force-choice options to choose from, including happiness, sadness, fear, disgust, anger, and surprise. The ERT scores are based on accuracy (number of correct/incorrect responses), and latency in response to particular stimuli, with a Cronbach’s alpha >0.70 previously reported [62].

### 2.6. Reading the Mind in the Eyes Test (RMET)

The RMET evaluates a component of affective theory of mind (aToM) referred to as mentalising [63,64]. The aToM refers to an individual’s capability to discern another person’s thoughts, intentions, and feelings and consequently respond accordingly in a social situation [63]. Furthermore, aToM corresponds closely with less executively demanding aspects of social cognition, such as emotional perception ability and emotion recognition through people’s facial expressions [65]. The RMET has 36 items, which assess mentalising, whereby participants are shown the eye region of the face and asked, untimed, to pick one of four options, which best describe the mental state. Items can be further grouped by positive, neutral, or negative valence [66,67]. The test includes a balanced combination of male and female images. The RMET has been found to be reliable and stable over time [68], with a Cronbach’s alpha of 0.88 [69].

### 2.7. Positive and Negative Symptom Scale (PANSS)

The PANSS is used clinically to evaluate positive, negative, and general psychopathology symptoms clinically associated with schizophrenia. The scale includes 30-items [70], with the following ratings (1 = absent, 2 = minimal, 3 = mild, 4 = moderate, 5 = moderate/severe, 6 = severe, 7 = extreme). The Cronbach’s alpha for the PANSS has a range of 0.70–0.85 [71].

### 2.8. Ethical Approval

Ethical approval was granted as part of the Immune Response and Social Cognition in Schizophrenia (iRELATE) project, by the Research Ethics Committee at the National University of Ireland Galway, University Hospital Galway, and at Tallaght Hospital Dublin. Initial funding for iRELATE was awarded through the European Research Council (ERC; PI: GD).

### 2.9. Statistical Analyses

The current study adopted a between-subjects design. IBM SPSS Statistics v25 was used to analyse previously mentioned neuroimaging and neuropsychological variables. The analysis was hypothesis-driven based on an evaluation of previous studies assessing WM variance and social cognition in patients with schizophrenia. As such, a multivariate analysis of variance (MANOVA), Pearson’s correlation, and linear regression (enter method) analysis were chosen as the most appropriate tests based on previous literature.

## 3. Results

### 3.1. Between-Group Diffusion Analysis

An analysis of variance (ANOVA) was conducted to compare whether significant FA differences were present in people with schizophrenia and healthy participants in this sample. The first analysis evaluated FA of ‘all WM tracts’ between typical controls and patients and revealed the expected significant difference (*F*_(1, 234)_ = 21.80, *p* < 0.001, η^2^ *p* = 0.085), such that patients showed reduced FA, compared to controls.

Following the above analysis, a further segmented multivariate analysis of variance (MANOVA) was conducted of the CC and its components. This revealed a significant difference between patients and typical participants within the CC (*F*_(1, 234)_ = 26.03, *p* < 0.001, η^2^ *p* = 0.10), as well as each of the subdivisions of the CC: the genu (*F*_(1, 234)_ = 27.82, *p* < 0.001, η^2^ *p* = 0.11); body (*F*_(1, 234)_ = 23.34, *p* < 0.001, η^2^ *p* = 0.09); and the splenium (*F*_(1, 234)_ = 12.26, *p* < 0.001, η^2^ *p* = 0.05). On all anatomical variables analysed, schizophrenia patients had reduced FA, when compared to typical controls for the CC, genu, body, and splenium. Mean scores and standard deviations of the patient and control participants can be found in Table 2 below.

The second MANOVA analysis used the variable ‘FA of all WM tracts’ as a covariate to assess its influence on the overall outcome. This analysis revealed a significant difference on the FA of the CC (*F*_(1, 233)_ = 4.55, *p* = 0.03, η^2^ *p* = 0.02), with the only sub-region of significance being the anterior region of the CC, the genu (*F*_(1, 233)_ = 6.15, *p* = 0.01, η^2^ *p* = 0.03). No significant difference remained for the body of the CC (*F*_(1, 233)_ = 3.26, *p* = 0.07, η^2^ *p* = 0.01) or the splenium of the CC (*F*_(1, 233)_ = 0.61, *p* = 0.43, η^2^ *p* = 0.01).

### 3.2. Social Cognition Comparisons

All social cognitive variables were assessed between healthy participants and schizophrenia patients using a MANOVA. The variables included in the first analysis were the RMET total, RMET positive valence percentage correct, RMET negative valence percentage correct, and RMET neutral valence percentage correct, as well as the overall median reaction time scores for the ERT, and overall *correct* reaction time scores for the ERT. The results of the analysis revealed a significant difference between groups for scores on the RMET total scores (*F*_(1, 223)_ = 28.81, *p* ≤ 0.001, η^2^ *p* = 0.11), positive valence (*F*_(1, 223)_ = 21.45, *p* < 0.001, η^2^ *p* = 0.09), negative valence (*F*_(1, 223)_ = 14.74, *p* < 0.001, η^2^ *p* = 0.06), and neutral valence (*F*_(1, 223)_ = 14.24, *p* < 0.001, η^2^ *p* = 0.06). There were also significant differences noted on the timed outcomes of the ERT, such as the overall median reaction time (*F*_(1, 223)_ = 31.50, *p* < 0.001, η^2^ *p* = 0.12), and overall median *correct* reaction time (*F*_(1, 223)_ = 32.10, *p* < 0.001, η^2^ *p* = 0.13). Typical controls performed better than patients on all social cognition outcomes. Means and standard deviations for the RMET and its subscales can be seen in Table 2.

A MANOVA was conducted to assess the total hits obtained on the ERT, i.e., happiness, sadness, fear, anger, surprise, and disgust. This analysis was conducted to measure accuracy. The results of the total hits analysis revealed a significant difference in total hits between schizophrenia patients and typical controls for total hits for happiness (*F*_(1, 300)_ = 35.18, *p* < 0.001, η^2^ *p* = 0.11), sadness (*F*_(1, 300)_ = 25.64, *p* < 0.001, η^2^ *p* = 0.08), fear (*F*_(1, 300)_ = 9.84, *p* < 0.001, η^2^ *p* = 0.03), anger (*F*_(1, 300)_ = 16.20, *p* < 0.001, η^2^ *p* = 0.05), surprise (*F*_(1, 300)_ = 17.90, *p* < 0.001, η^2^ *p* = 0.06), and disgust (*F*_(1, 300)_ = 54.40, *p* < 0.001, η^2^ *p* = 0.15), indicating typical controls performed better at correctly recognising emotional stimuli presented to them.

Reaction time on the ERT was further investigated for both correct and overall responding. Healthy controls were significantly faster at responding on the emotion recognition task overall (*F*_(1, 284)_ = 43.30, *p* < 0.001, η^2^ *p* = 0.13) and for each of the emotion stimuli: happiness (*F*_(1, 284)_ = 46.23, *p* < 0.001, η^2^ *p* = 0.14), sadness (*F*_(1, 284)_ = 15.65, *p* < 0.001, η^2^ *p* = 0.05), fear (*F*_(1, 284)_ = 4.51, *p* = 0.04, η^2^ *p* = 0.02), anger (*F*_(1, 284)_ = 15.23, *p* < 0.001, η^2^ *p* = 0.05), surprise (*F*_(1, 284)_ = 25.87, *p* < 0.001, η^2^ *p* = 0.08), and disgust (*F*_(1, 284)_ = 24.99, *p* < 0.001, η^2^ *p* = 0.08). A further MANOVA was conducted to assess *correct* reaction times with a significant difference between patients and typical controls noted on *correct* reaction time for happiness (*F*_(1, 284)_ = 25.87, *p* < 0.001, η^2^ *p* = 0.08), sadness (*F*_(1, 284)_ = 25.87, *p* < 0.001, η^2^ *p* = 0.08), fear (*F*_(1, 284)_ = 25.87, *p* = 0.01, η^2^ *p* = 0.08), anger (*F*_(1, 284)_ = 25.87, *p* = 0.01, η^2^ *p* = 0.08), surprise (*F*_(1, 284)_ = 25.87, *p* < 0.001, η^2^ *p* = 0.08), and disgust (*F*_(1, 284)_ = 25.87, *p* < 0.001, η^2^ *p* = 0.08) also. Means and standard deviations of median reaction time and *correct* reaction time can be viewed in Table 3.

### 3.3. Correlation

On account of the findings from the MANOVA analyses, a Pearson’s correlation was conducted with listwise deletion with schizophrenia patients only (*n* = 48) to investigate if microstructural integrity, as measured by FA related to outcomes on measures of social cognition. After controlling for multiple comparisons, there was a significant negative correlation found between median correct reaction time for happiness on the ERT and the FA of the anterior region of the CC, the genu (*r* = −0.367, *p* = 0.012). No significance was found between median correct reaction time for happiness and the body of the CC, or the splenium of the CC, following correction for multiple comparisons. These results indicate that reduced microstructural integrity, as measured by FA within the genu of the CC, is associated with longer reaction time to correctly identify happiness. No significant associations were found between anatomical regions of interest–genu, body, splenium, CC—and median correct reaction times for sadness, fear, anger, surprise, and disgust (*p* ≥ 0.05). Without controlling for correct responses, there were no associations found on overall median reaction time and the anatomical regions of interest (*p* > 0.05). This would suggest that the aforementioned association is not simply a result of reduced processing speed. Of clinical importance by comparison, no associations were found for healthy control participants (*p* > 0.05) between anatomical regions of interest (genu, body, splenium, or total CC) and reaction times (i.e., median reaction time or median correct reaction time for happiness, sadness, fear, anger, surprise, or disgust).

To evaluate clinical symptomatology, i.e., positive, negative, and general psychopathology symptoms on the PANSS, and the relationship with anatomical regions of interest, i.e., the CC and its subcomponents: genu, body, and splenium, an additional Pearson’s correlation was conducted for people with schizophrenia (*n* = 46). The analysis revealed significant and negative associations between the positive symptom scale and the CC (*r* = −0.301, *p* = 0.042). Sub-component correlations of the CC show that the genu of the CC (*r* = −0.293, *p* < 0.05) and splenium (*r*= −0.294, *p* < 0.05) but not the body of the CC (*r* = −0.248) are negatively and significantly corrected to the positive subscale of the PANSS. There were no significant associations noted for the negative or general PANSS scores and the anatomical regions of interest.

PANSS scores were also evaluated for associations with median *correct* reaction time variables (happiness, sadness, fear, anger, surprise, disgust). A Pearson’s correlation analysis was conducted on schizophrenia patients only (*n* = 46) and this analysis revealed no significant association between overall median correct reaction time or specific emotion reaction time and the PANSS subscales. Overall, the PANSS results indicate that the higher the positive symptom scores of patients, the lower FA in the anterior region of the CC.

### 3.4. Regression

Taking these clinical, cognitive, and anatomical findings together, a linear regression was conducted with the aforementioned patient group (n = 46) to see if the positive symptom scale of the PANSS was a predictor variable of the FA in the genu for people with schizophrenia. Following this, a second linear regression investigated whether FA of the genu was significantly associated with the ERT correct reaction time for happiness. Multicollinearity was not present in the data. The results of the regression analysis indicate that the positive symptom scale of the PANSS is significantly associated with the FA of the genu of the CC (*F*_(2, 45)_ = 4.12, *p* < 0.05, *R*^2^ = 0.086, adjusted *R*^2^= 0.065). Furthermore, it was shown that reduced FA of the genu was significantly associated with longer reaction times on the ERT for happiness (*F*_(2, 45)_ = 23.82, *p* < 0.001, *R*^2^ = 0.11, adjusted *R*^2^= 0.108). There was no direct predictive relationship observed between positive symptoms and reaction times on the ERT for happiness (*F*_(2, 45)_ = 0.241, *p* = 0.626, *R*^2^ = 0.005).

## 4. Discussion

This study aimed to investigate measures of social cognition and emotion recognition and their relationship to the structural integrity of the CC for people with schizophrenia relative to typical controls. This study observed reduced FA of WM tracts in the CC as a singular ROI, as well as its subregions: the genu, body, and splenium between patients and typical controls. Controlling for the overall difference in FA of all WM tracts, the main anatomical difference was evident in the genu of the CC, which was significantly related to positive symptomatology and poorer reaction time to correctly recognise emotions.

Specifically, people with schizophrenia’s reaction time to correctly identifying happiness was significantly and negatively associated with the FA in the genu of the CC after controlling for multiple comparisons. This could be for several reasons. One such reason may include reduced inter-hemispheric communication in people with schizophrenia. As the CC is a commissural fibre, which connects the two hemispheres, the results of this study could indicate reduced interhemispheric speed of information processing. In essence, the findings propose that how long it takes a person with schizophrenia to correctly identify, decode, recognise, and ultimately respond to a happy face (happiness), which is related to the structural integrity of the CC, and more specifically, the genu. Furthermore, this study established that the greater the positive symptom severity (i.e., hallucinations, delusions) in patients, the lower the FA in the genu of the CC.

Global and regional CC reductions in the genu [72], the body of the CC [73], and the splenium [72] has been established. According to a study by Koshiyama and colleagues [74], significant FA differences were identified in the CC and its subregions (the genu, body, and splenium), with similar findings showing decreased FA within the genu and body in a large sample of 287 people with schizophrenia and 193 typical controls [75]. Further studies have also identified specific and isolated abnormalities in the genu of the CC [76]. Moreover, a meta-analysis by Ellison-Wright and Bullmore [77] on chronic schizophrenia patients highlighted central anatomical regions where disconnection of WM were evident, which included the genu of the CC.

Notwithstanding, individuals at high risk of developing schizophrenia, through genetic predisposition, have also been found to have deficits in WM integrity, prior to the onset or development of clinical symptoms, with lower FA observed in the CC of those at clinical ultra-high risk [39]. Klauser et al. [78] demonstrated significant reduction in FA for schizophrenia patients within the CC, using a mediation analysis to examine if medication could explain the reduction in anisotropy; however, no differences were identified between medicated and unmedicated patients.

As part of the present study, clinical variables (i.e., symptom severity of schizophrenia) were evaluated. This finding is consistent with previous research, which found that decreased WM integrity in the CC was related to the severity of psychotic symptoms [79]. Our study is consistent with previous research showing social cognitive deficits in people with schizophrenia, compared with typical controls [80,81]. Patients had poorer recognition of emotional stimuli and also performed slower. This finding is in line with previous research. The fMRI studies have observed reduced activity within emotion recognition networks in people with a diagnosis of schizophrenia [82], and other studies have found that patients are worse at recognising emotions when compared with typical controls [12,83,84]. In the present study, schizophrenia patients showed a negative association between their reaction time to correctly identifying happiness and the structural integrity of the genu. The poorer the structural integrity within the genu of schizophrenia patients, the longer it took them to correctly identify happiness, which is in line with the work of Yang and colleagues [54].

Similarly within the literature, lower FA within the splenium correlates with poor social cognition scores [85]. Although this study had similar findings, it was not consistent following correction for multiple comparisons and correcting for the overall difference in WM FA. In line with our study, an earlier study by Zhao and colleagues [86] found reduced FA in the CC, which significantly correlated with emotion perception. Other studies have investigated the CC and clinical symptoms, though Fujino and colleagues [87] report significantly lower FA for schizophrenia patients, compared to typical controls, which negatively correlated with personal distress while controlling for positive and negative symptomology. Fujino and colleagues [87] concluded that reduced interhemispheric transference of information caused by disruption to the CC leads to longer interpretation time between information relating to the self and the environment.

## 5. Limitations and Future Research

There are some limitations to outline in this study. Although this study showed specific differences in white matter integrity between patients and controls, the data did not include specific left–right hemispheric tract breakdowns, where other studies have identified left–right differences of the CC [88]. Future research could replicate studies like the current study to include left–right hemispheric segmentations and investigate their link to social cognition. As this study was cross-sectional in its design, future studies could also focus on gathering longitudinal diffusion data and investigating fluctuations in both diffusion and cognitive profiles over time. Furthermore, repeated measurement of clinical symptomatology could determine whether reduced anisotropy is related to increased clinical symptoms, or vice versa, and larger sampling could allow for more robust multivariate analyses, such as structural equational modelling. As above, this study found that positive clinical symptomatology was associated with reduced structural integrity of the anterior region of the CC. This finding is of clinical importance as there is evidence showing that pharmacological intervention improves white matter integrity within the genu of the CC [56]; with other research showing psychological intervention improves frontal white matter tract integrity [89,90]. Consequently, building on the collective findings of this study, future research could investigate whether specific pharmacological or psychological intervention can improve white matter tract integrity for people with schizophrenia and whether that relates to a reduction in clinical symptomatology. This study also incorporated a whole group-based approach for our clinical cohort, which may be considered a limitation. Future research could, therefore, consider a more phenotypic approach to clinical, cognitive, and neuroimaging outcomes to further delineate the heterogenous profile associated with schizophrenia. Currently there is no specific, unified, agreed, or comprehensive theoretical or data-driven model of social cognition. As such, these findings outline a specific impairment, without a clear cognitive system to benchmark performance against. Clinicians, researchers, and future work should consider the development of a wholistic cognitive model, which, in turn, would allow for the advancement of more precise and individualized assessment and treatment paradigms for people, both with schizophrenia and without.

## 6. Conclusions

This study aimed to investigate social cognition and emotion recognition and how outcomes related to the integrity of WM tracts in people with schizophrenia, compared with typical controls. The main findings of this study show that people with schizophrenia have reduced microstructural integrity in the CC, inclusive of its subregions, the genu, body, and splenium. This reduced FA in the CC, specifically the genu, is significantly associated with increased positive clinical symptomatology i.e., hallucinations/delusions, while being negatively associated with reaction times for correctly identifying emotion, specifically happiness.

Findings in relation to poorer reaction times to emotion recognition could be due to reduced interhemispheric connectivity secondary to schizophrenia, with symptom severity significantly associated with reduced anisotropy in the genu of the CC. Future research could enhance these findings by investigating left and right hemispheric differences in people with schizophrenia, compared to typical controls. Additionally, future research could assess diffusion of the CC in patients alongside symptom severity over time. Better understanding of the mechanisms of cognitive dysfunction and the anatomical associations of clinical symptoms may lead to more structured and targeted cognitive and clinical interventions. This, in turn, may ameliorate difficulties associated with schizophrenia and reduce disabling occupational and social burden for people living with the condition.

## Figures and Tables

**Table 1:** Patient and control group characteristics.

Demographics	Patients (N = 58)	Typical Control (N = 178)
Age-M (SD)	42.40 (11.16)	36.11 (12.36)
Age Range	18–63 years	18–65 years
Gender (% Female)	30%	41%
Number of years in education	16.81 ± 3.90	14.94 ± 3.09
Currently Unemployment	59.6%	13.2%

**Table 2:** Means (M) and standard deviations (SD) for FA white matter regions of interest in schizophrenia patients (N = 58) and typical controls (N = 178).

		Typical Controls	Patients
Anatomical Variables	Range	M ± SD	M ± SD
Average FA (all tracts)	0–1	0.41 ± 0.01	0.40 ± 0.02
Corpus Callosum	0–1	0.71 ± 0.02	0.69 ± 0.03
Genu	0–1	0.68 ± 0.03	0.65 ± 0.04
Body	0–1	0.69 ± 0.03	0.66 ± 0.04
Splenium	0–1	0.75 ± 0.02	0.74 ± 0.03
Reading the Mind in the Eyes Total	0–36	27.24 ± 4.65	23.38 ± 4.43
Positive Valence % correct	0–100%	76.00 ± 13.82	65.25 ± 16.56
Negative Valence % correct	0–100%	72.57 ± 16.83	62.15 ± 17.22
Neutral Valence % correct	0–100%	80.65 ± 17.59	69.42 ± 21.59
Emotion Recognition Hits	0–48	30.80 ± 4.26	25.05 ± 6.56
Happy	0–8	6.80 ± 1.06	5.86 ± 1.68
Sad	0–8	5.62 ± 1.29	4.68 ± 1.87
Angry	0–8	3.66 ± 1.47	2.90 ± 1.68
Fearful	0–8	3.17 ± 1.90	2.46 ± 1.65
Surprise	0–8	6.00 ± 1.09	5.30 ± 1.74
Disgust	0–8	5.55 ± 1.66	3.90 ± 2.11

**Table 3:** Mean (M) and standard deviation (SD) of patients and controls on median reaction time and median correct reaction time in milliseconds.

	Typical Control		Patients	
	M	SD	M	SD
Happy: Median Reaction Time	1194.98	453.10	1714.30	835.71
Happy: Correct Reaction Time	1015.17	318.18	1382.95	525.30
Sadness: Median Reaction Time	1944.78	1037.70	2474.09	1078.85
Sadness: Correct Reaction Time	1562.30	869.44	2171.27	1049.31
Fear: Median Reaction Time	2487.24	1469.40	2873.57	1334.81
Fear: Correct Reaction Time	2183.56	1248.34	2610.16	1245.85
Anger: Median Reaction Time	1923.08	1061.20	2489.06	1292.80
Anger: Correct Reaction Time	1832.23	1106.13	2253.19	1172.52
Surprise: Median Reaction Time	1516.07	731.20	2020.77	875.63
Surprise: Correct Reaction Time	1319.92	574.95	1846.11	941.61
Disgust: Median Reaction Time	1965.17	1006.33	2651.70	1222.10
Disgust: Correct Reaction Time	1615.31	897.80	2419.36	1628.40

## References

[1] Laursen T.M., Nordentoft M., Mortensen P.B. (2014). Excess Early Mortality in Schizophrenia. Annu. Rev. Clin. Psychol..

[2] Legge S.E., Santoro M.L., Periyasamy S., Okewole A., Arsalan A., Kowalec K. (2021). Genetic Architecture of Schizophrenia: A Review of Major Advancements. Psychol. Med..

[3] Murphy C.E., Walker A.K., Weickert C.S. (2021). Neuroinflammation in Schizophrenia: The Role of Nuclear Factor Kappa B. Transl. Psychiatry.

[4] Radhakrishnan R., Skosnik P.D., Ranganathan M., Naganawa M., Toyonaga T., Finnema S., Hillmer A.T., Esterlis I., Huang Y., Nabulsi N. (2021). In Vivo Evidence of Lower Synaptic Vesicle Density in Schizophrenia. Mol. Psychiatry.

[5] Gillespie N.A., Kendler K.S. (2021). Use of Genetically Informed Methods to Clarify the Nature of the Association between Cannabis Use and Risk for Schizophrenia. JAMA Psychiatry.

[6] Ermakov E.A., Dmitrieva E.M., Parshukova D.A., Kazantseva D.V., Vasilieva A.R., Smirnova L.P. (2021). Oxidative Stress-Related Mechanisms in Schizophrenia Pathogenesis and New Treatment Perspectives. Oxid. Med. Cell. Longev..

[7] Rokita K.I., Dauvermann M.R., Mothersill D., Holleran L., Holland J., Costello L., Cullen C., Kane R., McKernan D., Morris D.W. (2021). Childhood Trauma, Parental Bonding, and Social Cognition in Patients with Schizophrenia and Healthy Adults. J. Clin. Psychol..

[8] Rog J., Błażewicz A., Juchnowicz D., Ludwiczuk A., Stelmach E., Kozioł M., Karakula M., Niziński P., Karakula-Juchnowicz H. (2020). The Role of GPR120 Receptor in Essential Fatty Acids Metabolism in Schizophrenia. Biomedicines.

[9] Tanaka M., Szabó Á., Spekker E., Polyák H., Tóth F., Vécsei L. (2022). Mitochondrial Impairment: A Common Motif in Neuropsychiatric Presentation? The Link to the Tryptophan–Kynurenine Metabolic System. Cells.

[10] Singh T., Poterba T., Curtis D., Akil H., Al Eissa M., Barchas J.D., Bass N., Bigdeli T.B., Breen G., Bromet E.J. (2022). Rare Coding Variants in Ten Genes Confer Substantial Risk for Schizophrenia. Nature.

[11] Tsuang M.T., Stone W.S., Faraone S. (2000). V Towards the Prevention of Schizophrenia. Biol. Psychiatry.

[12] Addington J., Addington D. (2008). Social and Cognitive Functioning in Psychosis. Schizophr. Res..

[13] Cicero D.C., Jonas K.G., Li K., Perlman G., Kotov R. (2019). Common Taxonomy of Traits and Symptoms: Linking Schizophrenia Symptoms, Schizotypy, and Normal Personality. Schizophr. Bull..

[14] Hoff A.L., Svetina C., Shields G., Stewart J., DeLisi L.E. (2005). Ten Year Longitudinal Study of Neuropsychological Functioning Subsequent to a First Episode of Schizophrenia. Schizophr. Res..

[15] Mothersill O., Tangney N., Morris D.W., McCarthy H., Frodl T., Gill M., Corvin A., Donohoe G. (2017). Further Evidence of Alerted Default Network Connectivity and Association with Theory of Mind Ability in Schizophrenia. Schizophr. Res..

[16] Mothersill D., King S., Holleran L., Dauvermann M., Patlola S., Rokita K., McManus R., Keynon M., McDonald C., Hallahan B. (2021). Interleukin 6 Predicts Increased Neural Response during Face Processing in a Sample of Individuals with Schizophrenia and Healthy Participants: A Functional Magnetic Resonance Imaging Study. NeuroImage Clin..

[17] Nyatega C.O., Qiang L., Adamu M.J., Younis A., Kawuwa H.B. (2021). Altered Dynamic Functional Connectivity of Cuneus in Schizophrenia Patients: A Resting-State FMRI Study. Appl. Sci..

[18] Beaudoin C., Beauchamp M.H. (2020). Social Cognition. Handbook of Clinical Neurology.

[19] Kennedy D.P., Adolphs R. (2012). The Social Brain in Psychiatric and Neurological Disorders. Trends Cogn. Sci..

[20] Mothersill O., Knee-Zaska C., Donohoe G. (2016). Emotion and Theory of Mind in Schizophrenia—Investigating the Role of the Cerebellum. Cerebellum.

[21] Battaglia S., Fabius J.H., Moravkova K., Fracasso A., Borgomaneri S. (2022). The Neurobiological Correlates of Gaze Perception in Healthy Individuals and Neurologic Patients. Biomedicines.

[22] Adolphs R. (2009). The Social Brain: Neural Basis of Social Knowledge. Annu. Rev. Psychol..

[23] Frith C.D. (2007). The Social Brain?. Philos. Trans. R. Soc. B Biol. Sci..

[24] Tsao D.Y., Livingstone M.S. (2008). Mechanisms of Face Perception. Annu. Rev. Neurosci..

[25] Kanwisher N., Yovel G. (2006). The Fusiform Face Area: A Cortical Region Specialized for the Perception of Faces. Philos. Trans. R. Soc. B Biol. Sci..

[26] Lieberman M.D. (2007). Social Cognitive Neuroscience: A Review of Core Processes. Annu. Rev. Psychol..

[27] Fett A.-K.J., Viechtbauer W., Penn D.L., van Os J., Krabbendam L. (2011). The Relationship between Neurocognition and Social Cognition with Functional Outcomes in Schizophrenia: A Meta-Analysis. Neurosci. Biobehav. Rev..

[28] Harvey P.D., Strassnig M.T., Silberstein J. (2019). Prediction of Disability in Schizophrenia: Symptoms, Cognition, and Self-Assessment. J. Exp. Psychopathol..

[29] Cowman M., Holleran L., Lonergan E., O’Connor K., Birchwood M., Donohoe G. (2021). Cognitive Predictors of Social and Occupational Functioning in Early Psychosis: A Systematic Review and Meta-Analysis of Cross-Sectional and Longitudinal Data. Schizophr. Bull..

[30] Couture S.M., Penn D.L., Roberts D.L. (2006). The Functional Significance of Social Cognition in Schizophrenia: A Review. Schizophr. Bull..

[31] Aghevli M.A., Blanchard J.J., Horan W.P. (2003). The Expression and Experience of Emotion in Schizophrenia: A Study of Social Interactions. Psychiatry Res..

[32] Mueller B.A., Lim K.O., Hemmy L., Camchong J. (2015). Diffusion MRI and Its Role in Neuropsychology. Neuropsychol. Rev..

[33] Canu E., Agosta F., Filippi M. (2015). A Selective Review of Structural Connectivity Abnormalities of Schizophrenic Patients at Different Stages of the Disease. Schizophr. Res..

[34] Holleran L., Kelly S., Alloza C., Agartz I., Andreassen O.A., Arango C., Banaj N., Calhoun V., Cannon D., Carr V. (2020). The Relationship between White Matter Microstructure and General Cognitive Ability in Patients with Schizophrenia and Healthy Participants in the ENIGMA Consortium. Am. J. Psychiatry.

[35] Ohoshi Y., Takahashi S., Yamada S., Ishida T., Tsuda K., Tsuji T., Terada M., Shinosaki K., Ukai S. (2019). Microstructural Abnormalities in Callosal Fibers and Their Relationship with Cognitive Function in Schizophrenia: A Tract-specific Analysis Study. Brain Behav..

[36] Kochunov P., Coyle T.R., Rowland L.M., Jahanshad N., Thompson P.M., Kelly S., Du X., Sampath H., Bruce H., Chiappelli J. (2017). Association of White Matter with Core Cognitive Deficits in Patients with Schizophrenia. JAMA Psychiatry.

[37] Szeszko P.R., Robinson D.G., Ashtari M., Vogel J., Betensky J., Sevy S., Ardekani B.A., Lencz T., Malhotra A.K., McCormack J. (2008). Clinical and Neuropsychological Correlates of White Matter Abnormalities in Recent Onset Schizophrenia. Neuropsychopharmacology.

[38] Rowland L.M., Spieker E.A., Francis A., Barker P.B., Carpenter W.T., Buchanan R.W. (2009). White Matter Alterations in Deficit Schizophrenia. Neuropsychopharmacology.

[39] Rigucci S., Santi G., Corigliano V., Imola A., Rossi-Espagnet C., Mancinelli I., De Pisa E., Manfredi G., Bozzao A., Carducci F. (2016). White Matter Microstructure in Ultra-High Risk and First Episode Schizophrenia: A Prospective Study. Psychiatry Res. Neuroimaging.

[40] Levine S.C., Banich M.T., Koch-Weser M.P. (1988). Face Recognition: A General or Specific Right Hemisphere Capacity?. Brain Cogn..

[41] Luh K.E., Rueckert L.M., Levy J. (1991). Perceptual Asymmetries for Free Viewing of Several Types of Chimeric Stimuli. Brain Cogn..

[42] Lee C.U., Shenton M.E., Salisbury D.F., Kasai K., Onitsuka T., Dickey C.C., Yurgelun-Todd D., Kikinis R., Jolesz F.A., McCarley R.W. (2002). Fusiform Gyrus Volume Reduction in First-Episode Schizophrenia: A Magnetic Resonance Imaging Study. Arch. Gen. Psychiatry.

[43] Onitsuka T., Niznikiewicz M.A., Spencer K.M., Frumin M., Kuroki N., Lucia L.C., Shenton M.E., McCarley R.W. (2006). Functional and Structural Deficits in Brain Regions Subserving Face Perception in Schizophrenia. Am. J. Psychiatry.

[44] Nazeri A., Chakravarty M.M., Felsky D., Lobaugh N.J., Rajji T.K., Mulsant B.H., Voineskos A.N. (2013). Alterations of Superficial White Matter in Schizophrenia and Relationship to Cognitive Performance. Neuropsychopharmacology.

[45] Karlsgodt K.H., van Erp T.G.M., Poldrack R.A., Bearden C.E., Nuechterlein K.H., Cannon T.D. (2008). Diffusion Tensor Imaging of the Superior Longitudinal Fasciculus and Working Memory in Recent-Onset Schizophrenia. Biol. Psychiatry.

[46] Liu X., Lai Y., Wang X., Hao C., Chen L., Zhou Z., Yu X., Hong N. (2013). Reduced White Matter Integrity and Cognitive Deficit in Never-Medicated Chronic Schizophrenia: A Diffusion Tensor Study Using TBSS. Behav. Brain Res..

[47] Pérez-Iglesias R., Tordesillas-Gutiérrez D., Barker G.J., McGuire P.K., Roiz-Santiañez R., Mata I., de Lucas E.M., Quintana F., Vazquez-Barquero J.L., Crespo-Facorro B. (2010). White Matter Defects in First Episode Psychosis Patients: A Voxelwise Analysis of Diffusion Tensor Imaging. Neuroimage.

[48] Del Re E.C., Bouix S., Fitzsimmons J., Blokland G.A.M., Mesholam-Gately R., Wojcik J., Kikinis Z., Kubicki M., Petryshen T., Pasternak O. (2019). Diffusion Abnormalities in the Corpus Callosum in First Episode Schizophrenia: Associated with Enlarged Lateral Ventricles and Symptomatology. Psychiatry Res..

[49] Cai Q., Van der Haegen L., Brysbaert M. (2013). Complementary Hemispheric Specialization for Language Production and Visuospatial Attention. Proc. Natl. Acad. Sci. USA.

[50] Alves N.T., Aznar-Casanova J.A., Fukusima S.S. (2009). Patterns of Brain Asymmetry in the Perception of Positive and Negative Facial Expressions. Laterality Asymmetries Body Brain Cogn..

[51] Hoppe K.D., Bogen J.E. (1977). Alexithymia in Twelve Commissurotomized Patients. Psychother. Psychosom..

[52] TenHouten W.D. (1986). Right Hemisphericity of Australian Aboriginal Children Ii: Conjugate Lateral Eye Movements. Int. J. Neurosci..

[53] Subramaniam K., Gill J., Fisher M., Mukherjee P., Nagarajan S., Vinogradov S. (2018). White Matter Microstructure Predicts Cognitive Training-Induced Improvements in Attention and Executive Functioning in Schizophrenia. Schizophr. Res..

[54] Yang M., Gao S., Zhang X. (2020). Cognitive Deficits and White Matter Abnormalities in Never-Treated First-Episode Schizophrenia. Transl. Psychiatry.

[55] Webster M.J., Knable M.B., Johnston-Wilson N., Nagata K., Inagaki M., Yolken R.H. (2001). Immunohistochemical Localization of Phosphorylated Glial Fibrillary Acidic Protein in the Prefrontal Cortex and Hippocampus from Patients with Schizophrenia, Bipolar Disorder, and Depression. Brain. Behav. Immun..

[56] Xiao Y., Sun H., Shi S., Jiang D., Tao B., Zhao Y., Zhang W., Gong Q., Sweeney J.A., Lui S. (2018). White Matter Abnormalities in Never-Treated Patients with Long-Term Schizophrenia. Am. J. Psychiatry.

[57] Yang C., Zhang W., Yao L., Liu N., Shah C., Zeng J., Yang Z., Gong Q., Lui S. (2020). Functional Alterations of White Matter in Chronic Never-treated and Treated Schizophrenia Patients. J. Magn. Reson. Imaging.

[58] Segal D.L. (2010). Diagnostic and Statistical Manual of Mental Disorders, Text Revision (DSM-IV-TR^®^).

[59] First M.B., Gibbon M. (2004). The Structured Clinical Interview for DSM-IV Axis I Disorders (SCID-I) and the Structured Clinical Interview for DSM-IV Axis II Disorders (SCID-II).

[60] Bodammer N., Kaufmann J., Kanowski M., Tempelmann C. (2004). Eddy Current Correction in Diffusion-weighted Imaging Using Pairs of Images Acquired with Opposite Diffusion Gradient Polarity. Magn. Reson. Med. An Off. J. Int. Soc. Magn. Reson. Med..

[61] Cantab C.C. (2016). Cognitive Assessment Software.

[62] Sirivichayakul S., Kanchanatawan B., Thika S., Carvalho A.F., Maes M. (2019). Eotaxin, an Endogenous Cognitive Deteriorating Chemokine (ECDC), Is a Major Contributor to Cognitive Decline in Normal People and to Executive, Memory, and Sustained Attention Deficits, Formal Thought Disorders, and Psychopathology in Schizophrenia Patients. Neurotox. Res..

[63] Baron-Cohen S., Wheelwright S., Hill J., Raste Y., Plumb I. (2001). The “Reading the Mind in the Eyes” Test Revised Version: A Study with Normal Adults, and Adults with Asperger Syndrome or High-Functioning Autism. J. Child Psychol. Psychiatry Allied Discip..

[64] Olderbak S., Wilhelm O., Olaru G., Geiger M., Brenneman M.W., Roberts R.D. (2015). A Psychometric Analysis of the Reading the Mind in the Eyes Test: Toward a Brief Form for Research and Applied Settings. Front. Psychol..

[65] Henry J.D., Phillips L.H., Beatty W.W., McDonald S., Longley W.A., Joscelyne A., Rendell P.G. (2009). Evidence for Deficits in Facial Affect Recognition and Theory of Mind in Multiple Sclerosis. J. Int. Neuropsychol. Soc..

[66] Carroll G.A., Montrose V.T., Burke T. (2021). Correlates of Social Cognition and Psychopathic Traits in a Community-Based Sample of Males. Front. Psychol..

[67] Carey E., Gillan D., Burke T., Burns A., Murphy T.M., Kelleher I., Cannon M. (2021). Social Cognition and Self-Reported ASD Traits in Young Adults Who Have Reported Psychotic Experiences: A Population-Based, Longitudinal Study. Schizophr. Res..

[68] Fernández-Abascal E.G., Cabello R., Fernández-Berrocal P., Baron-Cohen S. (2013). Test-Retest Reliability of the ‘Reading the Mind in the Eyes’ Test: A One-Year Follow-up Study. Mol. Autism.

[69] Israelashvili J., Sauter D., Fischer A. (2019). How Well Can We Assess Our Ability to Understand Others’ Feelings? Beliefs about Taking Others’ Perspectives and Actual Understanding of Others’ Emotions. Front. Psychol..

[70] Kay S.R., Fiszbein A., Opler L.A. (1987). The Positive and Negative Syndrome Scale (PANSS) for Schizophrenia. Schizophr. Bull..

[71] Van den Oord E.J.C.G., Rujescu D., Robles J.R., Giegling I., Birrell C., Bukszár J., Murrelle L., Möller H.-J., Middleton L., Muglia P. (2006). Factor Structure and External Validity of the PANSS Revisited. Schizophr. Res..

[72] Voineskos A.N., Farzan F., Barr M.S., Lobaugh N.J., Mulsant B.H., Chen R., Fitzgerald P.B., Daskalakis Z.J. (2010). The Role of the Corpus Callosum in Transcranial Magnetic Stimulation Induced Interhemispheric Signal Propagation. Biol. Psychiatry.

[73] Kubicki M., Park H., Westin C.-F., Nestor P.G., Mulkern R.V., Maier S.E., Niznikiewicz M., Connor E.E., Levitt J.J., Frumin M. (2005). DTI and MTR Abnormalities in Schizophrenia: Analysis of White Matter Integrity. Neuroimage.

[74] Koshiyama D., Fukunaga M., Okada N., Morita K., Nemoto K., Yamashita F., Yamamori H., Yasuda Y., Fujimoto M., Kelly S. (2018). Role of Frontal White Matter and Corpus Callosum on Social Function in Schizophrenia. Schizophr. Res..

[75] Oestreich L.K.L., Lyall A.E., Pasternak O., Kikinis Z., Newell D.T., Savadjiev P., Bouix S., Shenton M.E., Kubicki M., Bank A.S.R. (2017). Characterizing White Matter Changes in Chronic Schizophrenia: A Free-Water Imaging Multi-Site Study. Schizophr. Res..

[76] Dong D., Wang Y., Chang X., Jiang Y., Klugah-Brown B., Luo C., Yao D. (2017). Shared Abnormality of White Matter Integrity in Schizophrenia and Bipolar Disorder: A Comparative Voxel-Based Meta-Analysis. Schizophr. Res..

[77] Ellison-Wright I., Bullmore E. (2009). Meta-Analysis of Diffusion Tensor Imaging Studies in Schizophrenia. Schizophr. Res..

[78] Klauser P., Baker S.T., Cropley V.L., Bousman C., Fornito A., Cocchi L., Fullerton J.M., Rasser P., Schall U., Henskens F. (2017). White Matter Disruptions in Schizophrenia Are Spatially Widespread and Topologically Converge on Brain Network Hubs. Schizophr. Bull..

[79] Knöchel C., Oertel-Knöchel V., Schönmeyer R., Rotarska-Jagiela A., van de Ven V., Prvulovic D., Haenschel C., Uhlhaas P., Pantel J., Hampel H. (2012). Interhemispheric Hypoconnectivity in Schizophrenia: Fiber Integrity and Volume Differences of the Corpus Callosum in Patients and Unaffected Relatives. Neuroimage.

[80] Comparelli A., Corigliano V., De Carolis A., Mancinelli I., Trovini G., Ottavi G., Dehning J., Tatarelli R., Brugnoli R., Girardi P. (2013). Emotion Recognition Impairment Is Present Early and Is Stable throughout the Course of Schizophrenia. Schizophr. Res..

[81] Yalcin-Siedentopf N., Hoertnagl C.M., Biedermann F., Baumgartner S., Deisenhammer E.A., Hausmann A., Kaufmann A., Kemmler G., Mühlbacher M., Rauch A.-S. (2014). Facial Affect Recognition in Symptomatically Remitted Patients with Schizophrenia and Bipolar Disorder. Schizophr. Res..

[82] Jáni M., Kašpárek T. (2018). Emotion Recognition and Theory of Mind in Schizophrenia: A Meta-Analysis of Neuroimaging Studies. World J. Biol. Psychiatry.

[83] Kohler C.G., Walker J.B., Martin E.A., Healey K.M., Moberg P.J. (2010). Facial Emotion Perception in Schizophrenia: A Meta-Analytic Review. Schizophr. Bull..

[84] Turetsky B.I., Kohler C.G., Indersmitten T., Bhati M.T., Charbonnier D., Gur R.C. (2007). Facial Emotion Recognition in Schizophrenia: When and Why Does It Go Awry?. Schizophr. Res..

[85] Peng X., Zhang R., Yan W., Zhou M., Lu S., Xie S. (2020). Reduced White Matter Integrity Associated with Cognitive Deficits in Patients with Drug-Naive First-Episode Schizophrenia Revealed by Diffusion Tensor Imaging. Am. J. Transl. Res..

[86] Zhao X., Sui Y., Yao J., Lv Y., Zhang X., Jin Z., Chen L., Zhang X. (2017). Reduced White Matter Integrity and Facial Emotion Perception in Never-Medicated Patients with First-Episode Schizophrenia: A Diffusion Tensor Imaging Study. Prog. Neuro-Psychopharmacol. Biol. Psychiatry.

[87] Fujino J., Takahashi H., Miyata J., Sugihara G., Kubota M., Sasamoto A., Fujiwara H., Aso T., Fukuyama H., Murai T. (2014). Impaired Empathic Abilities and Reduced White Matter Integrity in Schizophrenia. Prog. Neuro-Psychopharmacol. Biol. Psychiatry.

[88] Park H.-J., Westin C.-F., Kubicki M., Maier S.E., Niznikiewicz M., Baer A., Frumin M., Kikinis R., Jolesz F.A., McCarley R.W. (2004). White Matter Hemisphere Asymmetries in Healthy Subjects and in Schizophrenia: A Diffusion Tensor MRI Study. Neuroimage.

[89] Tang Y.-Y., Lu Q., Geng X., Stein E.A., Yang Y., Posner M.I. (2010). Short-Term Meditation Induces White Matter Changes in the Anterior Cingulate. Proc. Natl. Acad. Sci. USA.

[90] Tang Y.-Y., Lu Q., Fan M., Yang Y., Posner M.I. (2012). Mechanisms of White Matter Changes Induced by Meditation. Proc. Natl. Acad. Sci. USA.

